# Can whole-drawer images measure up? A reply to Johnson et al. (2013)?

**DOI:** 10.3897/zookeys.500.9139

**Published:** 2015-04-27

**Authors:** John W.H. Trueman, David K. Yeates

**Affiliations:** 1 Research School of Biology, The Australian National University, Canberra, ACT 0200, Australia; 2 The Australian National Insect Collection, CSIRO National Research Collections Australia, PO Box 1700 Canberra ACT 2601

**Keywords:** Digitization, entomological collections, morphometrics, museum collections, dragonflies, Odonata

## Abstract

Johnson et al. (2013) found that morphometric measurements of dragonfly wings taken from actual specimens and measurements taken from whole-drawer images of those specimens were equally accurate. We do not believe that their conclusions are justified by their data and analysis. Our reasons are, first, that their study was constrained in ways that restrict the generalisability of their results, but second, and of far greater significance, their statistical approach was entirely unsuited to their data and their results misled them to erroneous conclusions. We offer an alternative analysis of their data as published. Our reanalysis demonstrates, *contra* Johnson et al., that measurements from scanned images are not a reliable substitute for direct measurement.

## Introduction

The use of whole-drawer imaging to rapidly digitize insect collections has been promoted in a recent special issue of this journal (#209, 2012). While various imaging technologies have been used (Blagoderov et al. 2012; Mantle et al. 2012; Bertone et al. 2012; Dietrich et al. 2012; Schmidt et al. 2012), all have the advantage of providing rapid digital access to the specimen holdings of entomological natural history collections. Remote curation is then possible, provided single dorsal specimen images are sufficient for identification. However, while whole-drawer imaging is a form of digitization, it is not a substitute for specimen databasing, and by itself produces images of groups of specimens that are not databased. Significant disadvantages to the method are (1) that the drawer images are not easily associated with the geocoded specimen data from the specimens contained in the drawers, and (2) the images represent a snapshot in time that will change when the drawers are curated and/or any specimens are added, removed or moved in the drawer. Most often label data is partly or entirely obscured by the insect above, further reducing the utility of whole-drawer images in specimen databasing initiatives. In addition, some of the imaging methods produce images with distortion and curvature around their edges.

We have no doubt that some of the challenges of using Satscan images in the curation of insect collections will be overcome by future technological and workflow improvements; however, we agree with Ang et al. (2013) that digitization efforts should only proceed if they enhance the quality and quantity of taxonomy, are feasible and have favourable cost-benefit ratios. For example, many, perhaps 20, expert international curation and research visits could be arranged for the equivalent cost of a Satscan device used in some collections for whole-drawer imaging, and many similar curation and research visits could be arranged for the same cost as the annual service contract and ongoing operational labour inputs.

Whole-drawer images could possibly be used for extracting morphometric measurements from the insects in the drawers, increasing their value in addressing scientific questions including taxonomic ones. In a recent edition of this journal, Johnson et al. (2013) compared three methods for taking morphometric measurements, specifically of wing length, from museum specimens of pinned and set insects. Their conclusion was that measurements taken from actual insects and those taken from whole-drawer images of specimens were equally accurate. Our reanalysis of their data, however, suggests that measurements from scanned images are not a reliable substitute for direct measurement.

It is generally accepted, in entomological collection practice, that the most accurate method for taking morphological measurements of a pinned insect is to excise the body part from the specimen, mount it on a microscope slide, and then measure it using a calibrated eyepiece or other micrometer. The advantage of slide mounting is that the body part is held flat and at the proper angle for taking the measurement. On the downside, slide mounting is a slow and resource-intensive process. Its greatest disadvantage, though, is that the specimen must be damaged if not destroyed. This disadvantage makes the slide-mount method unsuitable in many instances.

A quicker, and non-destructive method is to take measurements *in situ* using hand-held calipers. This usually involves temporarily removing the pinned specimen from its drawer and orienting it so the part to be measured is open to view. In modern practice the measurement typically is taken with a set of fine-tipped digital calipers. The advantages of this caliper method over the slide-mount method are that measurements are easier to take and the specimen need not be damaged. The perceived disadvantages are that a hand-held measurement may be less accurate and/or less repeatable than a slide-mount measurement, and that results might vary depending on who takes the measurements.

In recent years a new non-destructive method has become available. Digital scanning technology is now such that an undistorted, evenly-scaled digital images can be taken of an entire drawer of pinned insects (Beaman and Cellinese 2012). In principle, morphometric measurements could be taken from the scan. An obvious advantage of this method is that an entire collection could be scanned and the images held on file. The chief theoretical disadvantage is that lengths as measured from the scanned image might be on average too short, the issue being that unless the part to be measured is oriented precisely in the plane of the camera it will appear foreshortened in the image.

Johnson et al. set out to test whether the caliper method and scan method are acceptable alternatives to the slow, difficult and destructive slide-mount method. They measured the lengths of the right forewings in each of 71 pinned specimens of Odonata (dragonflies and damselflies), using first the scan method, then the caliper method, and finally the slide-mount method. Each wing was measured three times by each method. The same operator took all 639 measurements. Johnson et al. in fact ran two variants of the slide-mount method. In the first each slide was labeled with its specimen number. In the second the label was replaced with a randomly assigned code. The results did not differ. For simplicity we refer to the second version only. Nothing in our conclusions would be altered if the first version was used instead.

Johnson et al. calculated the average wing length of their sample insects under each method. The slide-mount method gave this average as 29.24 mm, the caliper method gave 29.38 mm and the scan method gave 28.77 mm. They calculated the standard error of each estimate. It was 1.04 mm under any method. Two correlation coefficients also were calculated, the first was between lengths estimated by the caliper method and the slide-mount method, the second was between lengths estimated by the scan method and the slide-mount method. These two correlation coefficients were then compared.

Johnson et al. argue that although the caliper method overstates the average length by 0.14 mm and the scan method understates it by 0.47 mm, each estimate lies within one standard error of the average length from the slide-mount method, and so each alternative method gives an acceptable measure of length. Likewise, there being no significant difference between the two correlation coefficients, they argue that both the caliper and the scan methods are equally accurate.

Why do we not accept these conclusions? Two relatively minor issues can be dealt with briefly. First, while a major concern with the caliper method is that it may lack repeatability across different practitioners, Johnson et al. did not address this issue. They showed only that one particular practitioner overestimated wing lengths by an average 0.14 mm. This single data point tells us very little. The study would need to be repeated several times by different practitioners before any general conclusion could be drawn.

Second, on examining their data on repeat measures within the scan method we observed a pattern that suggests a possible problem. We enquired of the corresponding author, and it transpires the scan method was not fully replicated. The scan was taken only once, with measurements being taken three times from the same image. Thus, Johnson et al. understate the variability or overestimate the repeatability of this method by leaving out measurement error associated with making the scan.

Our chief reason, however, for rejecting the conclusions that Johnson et al. came to, is not about these issues but instead concerns the statistical approach they took when analyzing their data. They applied statistical methods which would be appropriate only if every measurement were of an average-length wing and the only source of length variation across the sample was measurement error, a proposition patently not true of their data. From personal knowledge of the drawers of specimens on which their study was based, their specimens range in size from *Nannophya dalei* with wing length about 11 mm, to *Hemianax papuensis* at 47 mm. A majority of their specimens were from species of moderate size, say between 25 and 35 mm forewing length, but the average of all lengths in their sample refers to no species at all. The standard error of the estimate of an average length, the 1.04 mm which Johnson et al. use as their standard against which to judge the performance of the methods, is largely a result of some wings being long and others short. It has very little to do with measurement error. It is illogical to say, of these data, that a measurement method should be regarded as acceptable if it can produce an average wing length that lies within 1.04 mm (or should that be 2.08 mm?) of the true value. Johnson et al. make a similar error with the correlation coefficients. It should be no surprise that the correlations are similar, because no method is so poor that it mistakes a small wing for a large one. That their two correlation coefficients are not dissimilar in a standard statistical test for the difference between two correlation coefficients is almost entirely due to the sampled wings being of different sizes. Nothing about the efficacy of the measurement methods can be inferred from that statistic.

## Reanalysis

**Figure 1.**
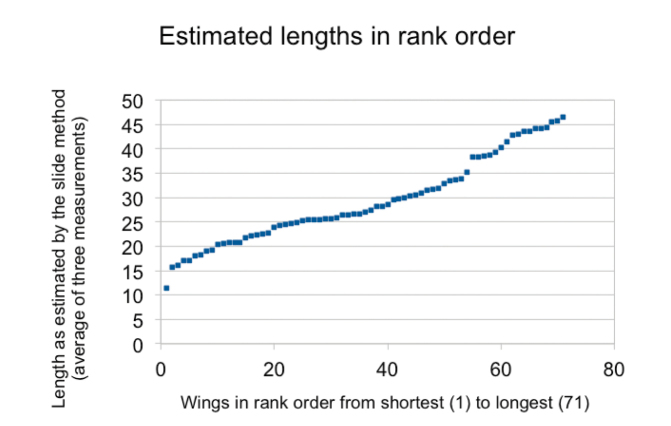
Averages (across the three repeat measurements) by the slide-mount method. Wings are arranged in size order.

**Figure 2.**
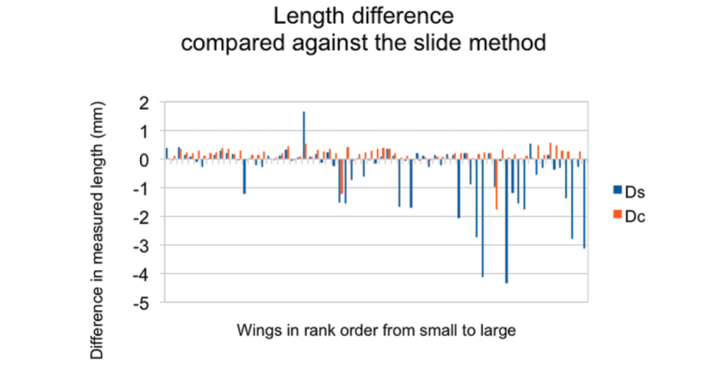
Averaged (across the three repeat measurements) length differences between pairs of methods. The order of the specimens is the same as for Figure 1. One series (Dc) is of differences between slide-mount and caliper lengths, the other (Ds) is between slide-mount and scan method lengths.

**Figure 3.**
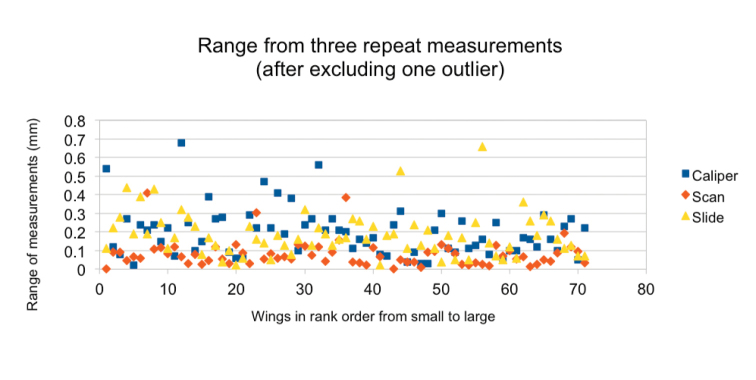
The range, in mm, for each wing by each method, specimens order being the same as before. Green symbols refer to the slide-mount method, blue to the caliper method and red to the scan method. One extreme outlier (ranked data point 42, specimen JT69) was removed.

Fortunately, Johnson et al. followed good practice and published their raw data in full. The analysis that follows takes the approach that comparisons ought to be made pairwise, wing by wing. The basic approach is that the three repeat measurements for each wing under each measurement method are averaged, and those three sets, each of 71 length estimates, are compared. We proceed by way of three related figures (Figures 1–3). In each figure the horizontal axis shows the 71 specimens arranged in size from small to large according to the slide-mount method.

The vertical axis in Figure 1 shows wing length. The message is that we have reasonable coverage of wings in the size range 15–45 mm. The several short ‘runs’ comprising a few wings of nearly identical size each represent, we may be fairly sure, one species. The vertical ‘gaps’, such as between 35 mm and 38 mm, represent lengths that are not sampled, quite possibly because no dragonflies in that size range occur where these specimens were collected.

Rather than compute the length of an ‘average’ wing, which is a biologically meaningless use of these data, let us note that the aggregate of all 71 measured lengths (averaged across the three repeat measurements) is 2076 mm by the slide-mount method, 2086 mm by the caliper method, and 2043 mm by the scan method. In other words, the caliper method, on average, has overstated the lengths by 0.48% (0.14 mm) while the scan method has understated them by 1.61% (0.47 mm) (using the slide-mount estimates as a reference length). These averaged differences or biases among the methods are, of course, exactly as reported by Johnson et al.

We might surmise that bias when using the caliper method might tend towards a fixed quantity that is independent of wing length. That would happen if the zero point of the calipers was wrongly set or the practitioner tended always to hold the instrument in some particular way that did not line up the instrument exactly with the specimen. Likewise, we might surmise that measurement bias in the scan method would tend towards a constant proportion. A constant percentage error would be expected, on averaging across many specimens, if the bias resulted primarily from some wings not being set in the horizontal plane.

Figure 2 shows differences in wing length; Dc represents the differences in length (averaged across three repeat measurements) between the caliper method and the slide-mount method, Ds represents the differences in length (similarly averaged) between the scan method and the slide-mount method. The differences in the first series are, indeed, quite uniformly distributed across all sizes of wing. Two large negative outliers, -1.22 mm (ranked data point 30, specimen JT63) and -1.77 mm (ranked data point 56, specimen JT60), drag the average down. Without access to the raw score sheets those scores cannot be verified but they look a lot like recording errors. On removing them from the calculation the average bias of the caliper method increases from +0.14 mm to +0.19 mm (+0.65%).

The second series (Ds) shows a pattern of frequent very large negative differences concentrated almost entirely in the large-winged half of the sample. This is in line with expectations if the main source of measurement error is foreshortening that affects some but not all specimens. One large positive outlier at ranked data point 24 (specimen JT33) does not fit any foreshortening explanation, and another outlier, ranked data point 14 (specimen JT20) appears very short in percentage terms and may also need separate explanation. As the figures stand, though, the scan method has understated the length in each of twenty specimens, being 28% of the sample, by more than 0.5 mm. The greatest difference, at -4.35 mm, is for ranked data point 58 (specimen JT19).

While this downward bias, expressed in absolute length difference, is greater for longer wings, long wings also show a higher proportionate bias. A least squares regression through the Ds scores (re-expressed as a percentage of wing length), and wing lengths by the slide-scan method, has a downward slope of 0.14% per millimetre of wing. This bias over and above what might be expected from foreshortening alone is explicable if, as is suggested by the Figure 2, a greater proportion of long wings than short wings are not exactly at right angles to the scanner. The average bias in the scan method as calculated from the regression would be close to 4% for a 46 mm wing. This average does not mean much, though, when in wings of every length the bias is concentrated in particular specimens.

It remains to examine each method for its repeatability. The ranges of the three repeat measurements can be used as an indicator. Fig. 3 shows the range of the three measurements, in mm, for each wing by each method.

There are no apparent trends in these intra-method repeatability statistics associated with wing size. Taking an average across the 71 observations, the slide-mount and caliper methods perform equally well. Averaged ranges are 0.185 mm (s.e. 0.119 mm) and 0.197 mm (s.e.0.130 mm) respectively. The difference between the observed means is not significant. At first sight the scan method appears to be more repeatable than either the caliper or the slide-mount method. The average range within the scan method is 0.083 mm after removing an outlier not shown in the figure (ranked data point 42, specimen JT69, range 2.03 mm). However, as established earlier, these data omit any error associated with repeating the scan, and so the comparison with the other methods is incomplete.

## Discussion and conclusion

Wings of various lengths within the range 11–47 mm have been measured by three methods, with sufficient coverage between 15 mm and 45 mm to give results that should be applicable within that range. The slide-mount method has been taken as a benchmark against which to compare the caliper method and the scan method. The sample (this from personal knowledge) was of typical drawers of pinned Odonata set by competent entomologists. The specimens were not of ‘show’ quality but neither were they of inferior quality. They were of a standard typically found in museum collections.

Using the caliper method, one practitioner has overestimated wing lengths by, on average, 0.19 mm. This bias was constant across the size range. The repeatability of the caliper method was similar to that of the slide-mount method, and the differences among repeat measurements are of similar size to the bias between the two methods. Further studies are needed to examine whether this bias and these levels of intra-method repeatability apply more broadly to other practitioners.

Using the scan method, the same practitioner underestimated wing lengths by, on average 0.47 mm. These errors were not constant across all wing sizes, and neither did they appear in all specimens. They were distributed erratically amongst some 28% of specimens and large errors, though not large percentage errors, occur almost exclusively in the long-winged half of the sample. Some of the errors were enormous; to >0.4 cm (and >10% of wing length) in the extreme case. The repeatability of this method has yet to be adequately examined.

The pattern of errors within the scan method contrasts with errors made by the same practitioner using the other methods. This suggests it is not an operator effect, and the limited information we have about scan measurement repeatability (which says it is similar to slide-mount and caliper repeatability only lower) confirms that conclusion. Rather, this pattern of errors is as would be expected under the hypothesis that downward bias occurs whenever a wing lies at an angle so that the image is foreshortened.

That this bias should apply to some 28% of specimens, and indeed to more than 40% of wings longer than about 25 mm in length, should be cause for concern. These data strongly suggest that the scan method is not suitable for use on larger insects. A method that can under-estimate in excess of 25% of wing lengths by more than a half millimetre, and at times produce errors of almost half a centimetre, is surely of little value as a measurement tool for entomologists. The method is not suitable for use on smaller insects either, because while a majority of wing lengths might be slightly underestimated, an occasional wing still is grossly underestimated by this method.

That said, if the technology of scanning could be improved to the point where out-of-plane wings could be recognised as such and the appropriate trigonometric corrections applied to measurements of the scanned image, the scan method might yet prove to contain an alternative to the other two methods.
